# The small molecule alpha-synuclein misfolding inhibitor, NPT200-11, produces multiple benefits in an animal model of Parkinson’s disease

**DOI:** 10.1038/s41598-018-34490-9

**Published:** 2018-11-01

**Authors:** Diana L. Price, Maya A. Koike, Asma Khan, Wolfgang Wrasidlo, Edward Rockenstein, Eliezer Masliah, Douglas Bonhaus

**Affiliations:** 1 Neuropore Therapies, Inc., San Diego, CA 92121 USA; 2 University of California, San Diego, Departments of Neuroscience and Pathology, La Jolla, CA 92093 USA; 3 Present Address: Vertex Pharmaceuticals, Inc., 11010 Torreyana Road, San Diego, CA 92121 USA

## Abstract

Accumulation of alpha-synuclein (ASYN) in neurons and other CNS cell types may contribute to the underlying pathology of synucleinopathies including Parkinson’s disease (PD), dementia with Lewy bodies (DLB) and Multiple Systems Atrophy (MSA). In support of this hypothesis for PD, ASYN immunopositive aggregates are a prominent pathological feature of PD, and mutations and gene multiplications of human wild type (WT) ASYN cause rare familial autosomal-dominant forms of PD. Targeted therapeutics that reduce the accumulation of ASYN could prevent or slow the neurodegenerative processes in PD and other synucleinopathies. NPT200-11 is a novel small molecule inhibitor of ASYN misfolding and aggregation. The effects of NPT200-11 on ASYN neuropathology were evaluated in animal models over expressing human alpha synuclein. Longitudinal studies using retinal imaging in mice expressing a hASYN::GFP fusion protein revealed that 2 months of once daily administration of NPT200-11 (5 mg/kg IP) resulted in a time-dependent and progressive reduction in retinal ASYN pathology. The effects of NPT200-11 on ASYN pathology in cerebral cortex and on other disease-relevant endpoints was evaluated in the Line 61 transgenic mouse model overexpressing human wild type ASYN. Results from these studies demonstrated that NPT200-11 reduced alpha-synuclein pathology in cortex, reduced associated neuroinflammation (astrogliosis), normalized striatal levels of the dopamine transporter (DAT) and improved motor function. To gain insight into the relationship between dose, exposure, and therapeutic benefit pharmacokinetic studies were also conducted in mice. These studies demonstrated that NPT200-11 is orally bioavailable and brain penetrating and established target plasma and brain exposures for future studies of potential therapeutic benefit.

## Introduction

Abnormal accumulation of misfolded alpha-synuclein (ASYN) has been hypothesized to underlie neuronal cell death and synaptic dysfunction in Parkinson’s disease (PD) and Dementia with Lewy Bodies (DLB). In support of this hypothesis, ASYN–containing intracellular inclusions (Lewy bodies and Lewy neurites) are a prominent pathological feature of PD^1^, and mutations and gene multiplications of human wild type (WT) ASYN cause rare familial autosomal-dominant forms of PD^2,3^. Targeted therapeutics which prevent the accumulation of ASYN in cell membranes could prevent or slow the neurodegenerative processes in PD and other synucleinopathies. Transgenic mouse models with overexpression of ASYN have proved useful in characterizing the behavioral, neuropathological, and biochemical consequences of ASYN aggregation^4^. Previous studies have demonstrated the beneficial effects of treatment *in vivo* with an ASYN misfolding inhibitor, NPT100-18A, on motor/sensorimotor behavior, and neuropathology endpoints in two different ASYN overexpressing transgenic mouse models of PD/DLB^5^.

NPT200-11, a novel compound with pharmacokinetic properties suitable for clinical evaluations, was developed with the aim of ameliorating PD-related symptoms and pathology by selectively inhibiting the misfolding of ASYN and subsequent accumulation. Here we present the results of *in vivo* pharmacodynamic imaging and efficacy evaluations of NPT200-11 activity utilizing transgenic mouse models of PD/DLB.

## Materials and Methods

### NPT200-11 compound

NPT200-11 was synthesized by Wuxi Apptec Co., Ltd. (Shanghai, China), and chemical purity was verified to be >95.9% via LC-MS. All other reagents were obtained from readily available commercial sources.

NPT200-11 and related compounds arose from a structure-based drug-discovery effort that utilized dynamic molecular modeling to identify and target specific regions of the alpha-synuclein protein critical for the formation of misfolded oligomers^5^. Initial lead compounds such as NPT100-18A demonstrated promising biological activities *in vitro* and in animal models, but had limited oral bioavailability, relatively poor brain penetration and other liabilities that precluded their advancement as therapeutic candidates. Lead-optimization efforts subsequently yielded NPT200-11, which retained the ability to inhibit alpha-synuclein misfolding *in vitro* (J. Wong, Neuropore Therapies, *personal communication, manuscript in preparation)* with substantially improved physiochemical and pharmacokinetic properties (see Supplemental Materials – for comparison of key mouse pharmacokinetic parameters for NPT100-18A and NPT200-11).

### Pharmacokinetic studies in wildtype C57BL/6 mice

Pharmacokinetic studies were performed to determine the plasma and brain distributions of NPT200-11 in male C57BL/6 mice following a single 10 mg/kg intravenous (IV), intraperitoneal (IP) or oral (PO) dose of NPT200-11. Mouse pharmacokinetic evaluations were performed by Sai Life Sciences Limited (Pune, India) in accordance with guidelines of the Institutional Animal Ethics Committee (IAEC). Three mice per route of administration at nine time points were assessed for a total of 81 mice (for IV and IP routes = pre-dose, 0.08, 0.25, 0.5, 1, 2, 4, 8, and 24 hours; and for PO route = pre-dose, 0.25. 0.5, 1, 2, 4, 6, 8 & 24 hours).

### Treatment regimen for *in vivo* imaging and efficacy studies

NPT200-11 was dissolved in a vehicle solution consisting of 40% Captisol in sterile water, and administered at a volume of 0.1 ml/20 g of body weight. Animals received a Monday-Friday daily intraperitoneal injection of vehicle, 0.5, 1 or 5 mg/kg NPT200-11 for approximately 90 days. Solutions were blind coded and experimenters were blinded to treatment for the duration of studies. Animals received treatment up to and including a final injection 1 hr prior to euthanasia.

### Longitudinal retinal imaging of GFP tagged ASYN in the PDNG78-alpha-synuclein transgenic mouse

We previously reported the development of a non-invasive live imaging assay to enable longitudinal studies of the effects of therapeutic intervention on ASYN accumulation in the retina of mice overexpressing fused ASYN-eGFP (ASYN::GFP) under the PDGF-beta promoter (PDNG78 line)^6^. The PDNG78 transgenic mouse line develops biochemical and neuropathological features consistent with Dementia with Lewy Bodies (DLB)/Parkinson’s disease (PD)^7^ and has been used previously for imaging ASYN in the CNS^8,9^ and retina^6^. In the latter report, retinal imaging at four separate time points over a three month period revealed accumulation of GFP tagged ASYN in retinal ganglion cells, and that this accumulation of ASYN persisted in the same cells over time and increased with age, demonstrating that retinal imaging in the PDNG78 line could be a useful tool to monitor ASYN accumulation and evaluate treatment interventions targeting ASYN in the CNS in a non-invasive manner. A dose of 5 mg/kg NPT200-11 was chosen for evaluation in the PDNG78 transgenic mice based on neuropathological findings of decreased levels of ASYN deposits in 1 month pilot efficacy studies utilizing the Line 61 transgenic mouse (*data not shown*).

Retinal imaging was performed at the University of California at San Diego (UCSD) under an amendment to UCSD Institutional Animal Care and Use Committee (IACUC) Protocol S02221. UCSD is an Institutional Animal Care and Use Committee accredited institution and the Animal Subjects Committee approved the experimental protocol followed in all studies according to the Association for Assessment and Accreditation of Laboratory Animal Care International guidelines. A total of 11 non-transgenic and 16 PDNG78 transgenic mice were utilized for the longitudinal imaging evaluations starting at 3 months of age. A Phoenix Micron III Retinal Imaging Microscope (Phoenix Research Labs, Pleasanton, CA) was utilized for non-invasive bright-field and fluorescent retinal imaging studies in anesthetized ASYN::GFP transgenic and non-transgenic mice, and the imaging and analysis was performed as previously described^6^. A baseline imaging evaluation was performed prior to starting daily administrations of vehicle or 5 mg/kg NPT200-11, followed by imaging at one month intervals for three months (total of 4 imaging sessions per mouse).

### 3 month efficacy evaluations in the Line 61 alpha-synuclein transgenic mouse model of Parkinson’s disease

Two separate three-month chronic administration studies using the mThy1-alpha-synuclein transgenic mouse model of PD with an overlapping 1 mg/kg dose of NPT200-11 are presented herein as combined data set. Heterozygous male transgenic mice and their non-transgenic littermates (approximately 3–3.5 months of age) were utilized for these studies. These studies were conducted by Neuropore Therapies employees at a vivarium facility at UCSD under UCSD Institutional Animal Care and Use Committee (IACUC) Protocol S02221 and the Animal Subjects Committee approved the experimental protocol followed in all studies according to the Association for Assessment and Accreditation of Laboratory Animal Care International guidelines.

This transgenic mouse model (commonly referred to as Line 61 transgenic mice^10^), overexpresses wild-type human ASYN under the murine Thy-1 promoter and develops extensive accumulation of ASYN in areas relevant to PD^11–13^, neurodegeneration (including loss of tyrosine hydroxylase immunoreactivity in the striatum^14^), inflammation^7,11,15^, and both motor and non-motor functional deficits^16,17^.

### Behavioral evaluations for 3 month efficacy studies in Line 61 transgenic mice

Behavioral assessments were started on approximately day 70 of treatment, between the hours of 09:00 and 16:00. All behavioral assessments were conducted by Neuropore employees in a vivarium facility at UCSD.

All behavioral data were analyzed by one-way ANOVA, first to test for a phenotypic difference between vehicle-treated groups and then to test for treatment effects of NPT200-11 administration in Line 61 ASYN transgenic mice. In the event of a statistically significant ANOVA, *post hoc* comparisons were made using Dunnett’s multiple comparisons test with the vehicle-treated Line 61 ASYN transgenic group as the control. The criterion for statistical significance was *p* < 0.05 for both analyses. Outlier data points were analyzed using Grubb’s method of outlier analysis, and applied to all groups for that measure. The data are presented as the mean ± the standard error of the mean (SEM).

#### Grip strength evaluation

Line 61 ASYN transgenic mice have robust grip strength deficits compared to age-matched non-transgenic littermates as early as 3 months of age (*unpublished observations*). Grip strength evaluations were conducted at baseline (prior to commencing daily treatments) and used primarily to pseudorandomize the assignment of treatment groups (*i.e*., ensure that each group had an equal distribution of grip strength values). Subjects were also re-tested after approximately 70 days of treatments as an additional motor performance measure.

Peak grip strength data were collected using a grip strength apparatus from San Diego Instruments (Poway, CA), which consists of an adjustable mesh grip with an integrated force gauge. This test is used to quantify an animal’s active resistance to being pulled off a horizontal bar. The (chatillion) force gauge was set to record peak fore limb strength in grams force. Grasping the animal’s tail at the base, the animal was placed just above the wire, and lowered until animal grabbed the wire with both front feet. The experimenter then gently pulled backward until animal released its grip on the wire. Each animal was tested in 5 consecutive trials, with a brief period of rest in between each trial. If the animal bit the wire, failed to grab with both front feed or used his hind limbs, the data from that trial were excluded. The dependent measure, peak strength (in grams of force), was determined per trial and as an average for each animal across trials.

#### Round beam traversal test

The round beam traversal test was performed as a combined measurement of gait and balance, and the Line 61 transgenic mice have been previously shown to exhibit deficits on this test^5^. Round beam data were collected using a custom built apparatus consisting of two 1 meter long removable acetel plastic rods (3 and 1 cm diameter) on a smooth acrylic frame elevated 17.5 to 22.5 cm above a testing bench. The beams were periodically roughed with 80 grit sand paper. The unit was wiped clean with laboratory wipes and water in between tested animals, and with Rx 44 HDQ (a one-step disinfectant solution) at the end of each day.

Each animal was trained by three consecutive trials on the 3 cm diameter beam and then tested on the 1 cm diameter beam with a brief break in between each trial. Each foot slip (past the midpoint of the beam) was counted by the experimenter blinded to treatment group. Any animal that did not stay on the apparatus for at least 5 seconds before falling was excluded from the analyses. The session ended when the animal completed the transit of the beam or at a 60 second cutoff. Test trials were recorded for secondary verification using a video recording system. The dependent measure of performance was the number of foot slips while on the beam.

### Analysis of NPT200-11 exposures in plasma and brain of transgenic mice

The development of a bioanalytical method and subsequent sample processing for analysis of NPT200-11 exposure levels in plasma and brain homogenate samples from subjects of the 3 month efficacy evaluations in Line 61 ASYN transgenic mice were conducted by MicroConstants, Inc. (San Diego, CA*)*. The calibration curves were conducted using blank mouse plasma and brain matrices provided by Neuropore Therapies, Inc. Group data are presented as mean concentrations of NPT200-11 (ng/ml) ± standard error of the mean (SEM).

### Neuropathological evaluations for 3 month efficacy study

Tissue collection, processing, and imaging methods were conducted as described previously^13^ and performed under a lab service agreement between UCSD and Neuropore Therapies.

All subjects were euthanized within 1 hour of the last treatment and brain and blood were collected. The right hemibrain was post-fixed in phosphate-buffered 4% PFA (pH 7.4) at 4 °C for 48 and then serially sectioned into 40 uM thick sagittal sections using a vibratome. Sections were free-floated and incubated overnight at 4 °C with primary antibodies. To confirm the specificity of primary antibodies, control experiments were performed in which sections were incubated overnight in the absence of primary antibody, preimmune serum, or primary antibody pre-adsorbed for 48 h with 20-fold excess of the corresponding peptide.

Statistical analyses for neuropathological data were performed using separate one-way analysis of variance (ANOVA) tests (Prism Graph Pad Software, San Diego, CA, USA) of specific pairwise comparisons determined *apriori*. In the event of a significant ANOVA, *post hoc* comparisons were made using Dunnett’s multiple comparisons test with the transgenic/vehicle group as the control. The significance level was set at p < 0.05. The data are expressed as group mean values ± standard error of the mean (SEM).

Cortical ASYN immunolabeling was conducted using a monoclonal anti-ASYN antibody (SYN-1, Clone 42; 1:500; #610787, BD Biosciences, San Diego, CA). This antibody recognizes a conserved epitope in human and rodent localized to residues 91–99 of the full length ASYN which overlaps portions of the NAC and C-terminus regions of the protein and therefore recognizes both wildtype and post translationally modified forms of ASYN^18^. Immunolabeling of total ASYN in cortex was evaluated as a corrected optical density across a field of view, as well as by counts of immunopositive cell bodies. A separate set of sections were pretreated with proteinase K (PK+) (#3115879001, Sigma, St. Louis, MO) prior to a blocking step to isolate insoluble forms of ASYN prior to immunolabeling. Immunolabeling of PK + resistant ASYN in cortex was evaluated as corrected topical density across a field of view.

Immunolabeling studies of other neurodegeneration-relevant markers utilized antibodies (EMD Millipore, Temecula, CA) against tyrosine hydroxylase (1:1000, MAB5280), NeuN (1:1000, ABN78), glial fibrillary acidic protein (GFAP; 1:500, AB5804), and dopamine transporter (DAT; 1:250, MAB369). Evaluations of tyrosine hydroxylase, GFAP, and DAT were evaluated and are presented as corrected optical densities across a field of view. Evaluations of tyrosine hydroxylase in the substantia nigra and NeuN were conducted and presented as counts of immunopositive cell bodies.

Sections were incubated with the primary antibody followed by incubation with biotinylated secondary antibodies (Vector Laboratories, Burlingame, CA) and visualized using an avidin-biotin (ABC) kit (Vector Laboratories, Burlingame, CA) with diaminobenzidine tetrahydrochloride (DAB; Sigma-Aldrich, St. Louis, MO) as the chromogen. Analyses were performed on blind-coded sections from transgenic and non-transgenic mice, as described previously^13^.

## Results

### Mouse pharmacokinetic studies

Pharmacokinetic parameters of NPT200-11 in plasma and brain following a single 10 mg/kg intravenous, intraperitoneal or oral administration in male C57BL/6 mice are summarized in Table 1. Following intravenous administration of NPT200-11 to C57BL/6 mice, NPT200-11 exhibited high systemic plasma clearance (80.68 mL/min/kg, where normal liver blood flow in mice is 90 mL/min/kg) with a terminal elimination plasma half-life (T_1/2_) of 1.11 hr. The volume of distribution of NPT200-11 was 5-fold higher than the normal volume of total body water (0.7 L/kg), indicating extravascular distribution. The brain to plasma exposure (AUC_last_) ratio was 1.99. After oral administration of NPT200-11 to C57BL/6 mice, the T_max_ in plasma and brain was observed to be 0.50 and 0.25 hr, respectively. The brain to plasma exposure (AUC_last_) ratio was 0.44. The oral bioavailability of NPT200-11 was found to be 53% (see Supplementary Table S1 for comparison to key mouse pharmacokinetic properties of NPT100-18A). After intraperitoneal administration of NPT200-11 to C57BL/6 mice, the T_max_ in plasma and brain was observed to be 0.25 and 0.50 hr, respectively. The brain to plasma exposure ratio (AUC_last_) was 1.05.

**Table 1:** Pharmacokinetic parameters of NPT200-11 in plasma and brain following a single 10 mg/kg intravenous, intraperitoneal or oral administration in male C57BL/6 mice.

Route	Matrix	T_max_ (hr)	C_max_ (ng/mL)	AUC_last_ (hr*ng/mL)	T_1/2_ (hr)	Clearance (mL/min/kg)	Volume of distribution (L/kg)	Brain to plasma exposure ratio	%F
IV	Plasma	—	2223.04	2061.89	1.11	80.68	3.62	1.99	
	Brain	—	5279.62	4107.48	1.75	40.13	2.28		
PO	Plasma	0.5	891.09	1092.54	—	—	—	0.44	53
	Brain	0.25	348.31	482.07	—	—	—		
IP	Plasma	0.25	1022.17	1528.71	—	—	—	1.05	
	Brain	0.5	1291.82	1611.21	—	—	—		

Notes:

1 AUC_last_ considered for calculating bioavailability.

2 The density of brain homogenate was considered as 1 which is equivalent to plasma density.

**Figure 1:**
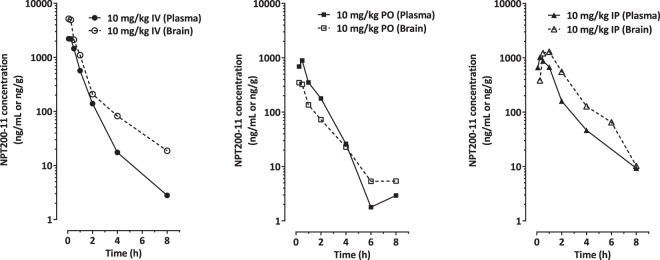
Brain levels of NPT200-11 parallel plasma levels following all routes of administration in C57BL/6 mice. Compound exposure data are presented as group means for specific time points (*from left to right*) for intravenous, oral, and intraperitoneal administration.

The data indicate that orally-administered NPT200-11 is well-absorbed in mice and distributes readily into brain tissue. Moreover, brain and plasma exposures were in parallel following all routes of administration (Fig. 1). These results compared favorably to the prototype compound, NPT100-18A (Brain to plasma ratio of 0.03 and an oral bioavailability of 0.32% in mice (see Supplementary Table S1 for additional mouse pharmacokinetic parameters compared).

### Retinal imaging of ASYN::GFP in PDNG78 ASYN transgenic mice

**Figure 2:**
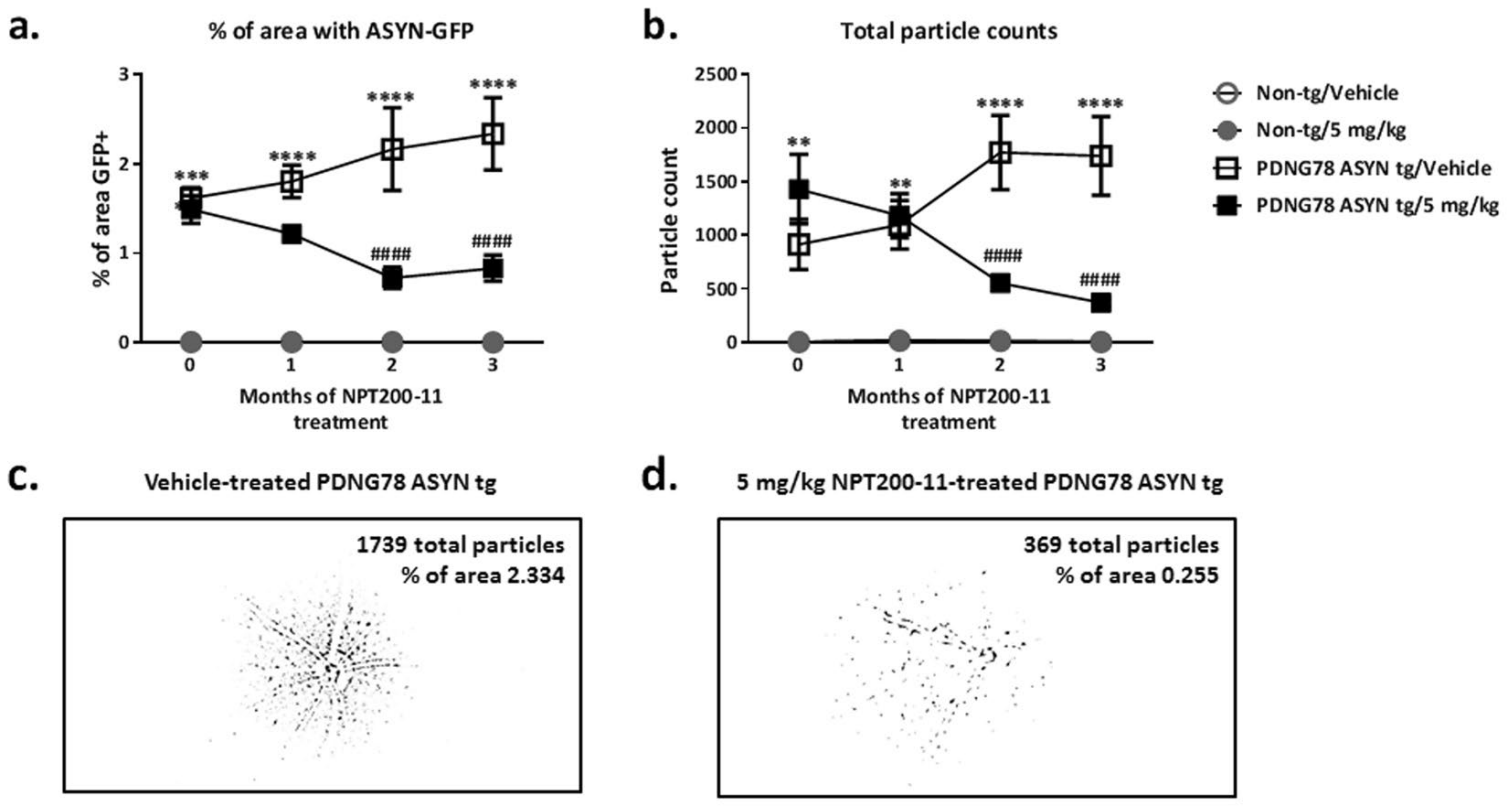
Three months of NPT200-11 treatment reduces ASYN::GFP in retinae of PDNG78 ASYN transgenic mice. Analysis of ASYN::GFP as (**a**) percentage of area and (**b**) total particle counts revealed statistically significant increased levels of ASYN::GFP at baseline (0, prior to commencement of treatments) in PDNG78 ASYN transgenic mice (**p < 0.01 and ***p < *0.001 vs. vehicle-treated non-transgenic control mice*) with persistent and/or progressive increases in vehicle-treated PDNG78 transgenic mice at subsequent imaging sessions (**p < 0.01 and ****p < *0.0001 vs. vehicle-treated non-transgenic control group*). Statistically significant treatment effects of NPT200-11 (5 mg/kg) emerged at the 2 month imaging session for both measures of ASYN::GFP, and persisted through the evaluation at 3 months of treatment (^*####*^*p* < *0.0001 vs. vehicle-treated PDNG78 ASYN transgenic control group*). Representative thresholded binary images are shown for (**c**) vehicle-treated and (**d**) NPT200-11-treated PDNG78 ASYN transgenic mice after 3 months of treatment.

Analysis of retinal images revealed statistically significant increases in ASYN::GFP levels as a (Fig. 2a) *percentage of area* and (Fig. 2b) *total particle counts* in PDNG78 transgenic mouse retina at baseline (**p < *0.01 and* ***p < *0.001 vs. vehicle-treated non-transgenic control mice*) as well as the next three imaging sessions at approximately 30 day intervals (**p < *0.01 and* ****p < *0.0001 vs. vehicle-treated non-transgenic control group*). Statistically significant treatment effects of NPT200-11 (5 mg/kg) emerged at the 2 month imaging session (treatment day 60) for both measures of ASYN::GFP, and persisted through the evaluation at 3 months of treatment (^***####***^*p* < *0.0001 vs. vehicle-treated PDNG78 ASYN transgenic control group*).

### 3 month efficacy evaluations in the Line 61 alpha-synuclein transgenic mouse model of Parkinson’s disease

#### Bioanalytical determination of NPT200-11 exposure levels in plasma and brain homogenates

**Figure 3:**
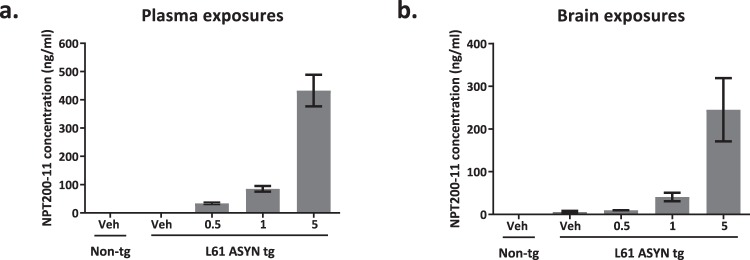
Bioanalysis of NPT200-11 exposures in non-transgenic and Line 61 ASYN transgenic mouse plasma and brain homogenates from the 3 month efficacy study. Genotype and treatment group (vehicle or NPT200-11 in mg/kg) are denoted along the x-axis. Graphed values represent the mean concentration of NPT-200-11 (ng/ml) ± SEM for 11–25 animals per group. The group statistics are summarized in Table 2.

Analysis of NPT200-11 exposures in plasma and brain of Line 61 ASYN transgenic mice confirmed detectable amounts of NPT200-11 in plasma and brain homogenates study samples (Fig. 3 and Table 2). The plasma and brain NPT200-11 exposure values in Line 61 ASYN transgenic mice were roughly linear and are in line with those obtained in wildtype mice

**Table 2:** Summary of NPT200-11 exposure bioanalysis of plasma and brain homogenates from Line 61 transgenic efficacy study.

Matrix	Non-tg/vehicle	Line 61 ASYN tg/vehicle	Line 61 ASYN tg/0.5 mg/kg	Line 61 ASYN tg/1 mg/kg	Line ASYN tg/5 mg/kg
Plasma (ng/mL)	0 ± 0	0.2452 ± 0.2452	33.95 ± 3.931	85.19 ± 10.07	432.8 ± 56.11
Brain (ng/mL)	0 ± 0	5.98 ± 2.197	9.709 ± 0.7619	40.96 ± 10.04	245.1 ± 73.89

*Exposure data are presented as group means* ± *standard error of mean for plasma and brain homogenates*.

#### Behavioral evaluations for 3 month efficacy studies

**Figure 4:**
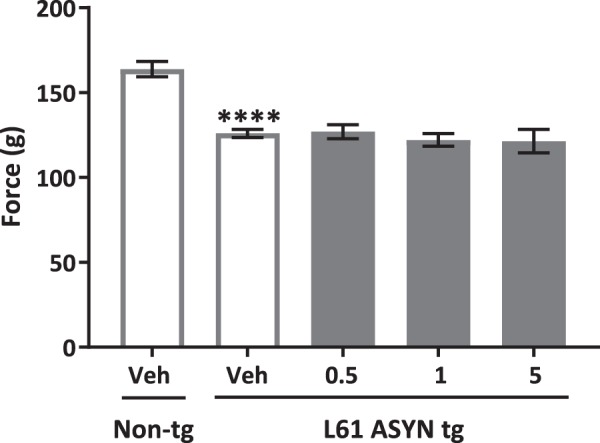
No effect of NPT200-11 treatment on Line 61 ASYN transgenic grip strength. There were statistically significant decreases in vehicle-treated Line 61 ASYN transgenic mouse grip strength (****p < *0.0001 vs. vehicle-treated non-transgenic control mice)*, that were not improved by NPT200-11-treatment at any dose.

Grip strength evaluation: Evaluation of grip strength demonstrated a statistically significant decrease in peak force values (Fig. 4) for grip strength in vehicle-treated Line 61 ASYN transgenic mice (****p < *0.0001 vs. vehicle-treated non-transgenic control group*). There were no statistically significant effects of NPT200-11 treatment on Line 61 ASYN transgenic grip strength.

**Figure 5:**
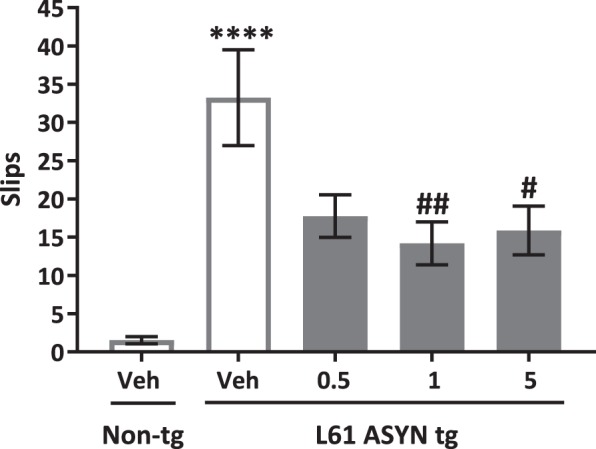
NPT200-11 treatment improves Line 61 ASYN transgenic round beam performance. There was a statistically significant increase in the number of slips in vehicle-treated Line 61 ASYN transgenic control mice on the round beam performance test (****p < *0.0001 vs. non-tg/vehicle control group*). There were statistically significant decreases in slips in ASYN transgenic mice treated with either 1 or 5 mg/kg NPT200-11 (^*##*^*p* < *0.01* and ^*#*^*p* < *0.05 vs. vehicle-treated Line 61 ASYN transgenic mice, respectively)*.

Round beam traversal test: Vehicle-treated Line 61 ASYN transgenic mice were impaired in round beam traversal performance (Fig. 5) as indicated by a statistically significant increase in slips as they traversed the apparatus (****p < *0.0001 vs. vehicle-treated non-transgenic control mice*). NPT200-11 (1 & 5 mg/kg) improved Line 61 ASYN transgenic performance on the round beam as indicated by a statistically significant decrease in the number of slips as they traversed the round beam (^*##*^*p* < *0.01 and* ^*#*^*p* < *0.05 vs. vehicle-treated Line 61 ASYN transgenic control group*).

#### Evaluations of Neuropathology

**Figure 6:**
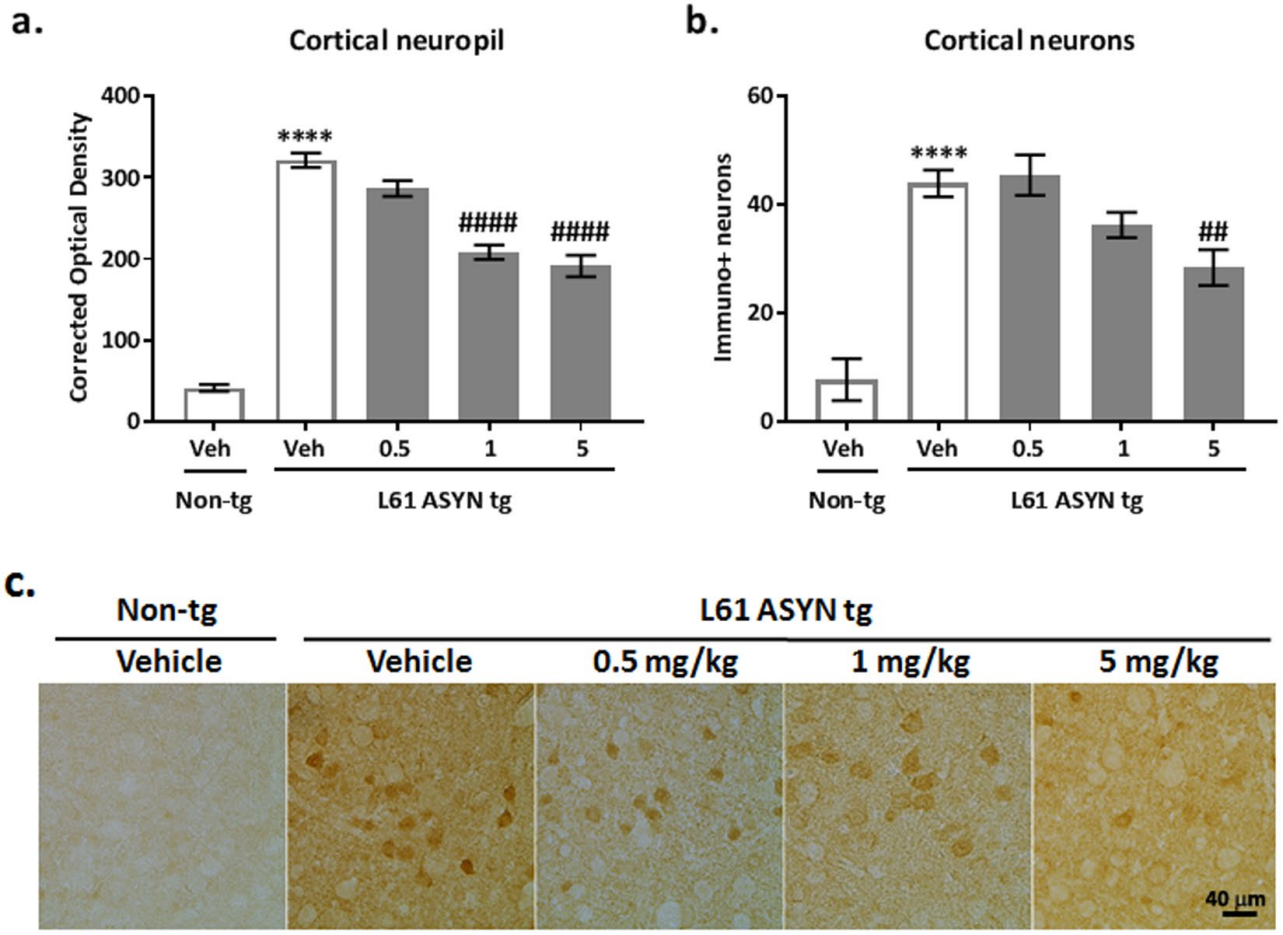
NPT200-11 administration decreased ASYN immunolabeling of cortical neuropil and neurons in the Line 61 ASYN transgenic mouse. There was a statistically significant increase in (**a**) ASYN immunolabeling in the cortical neuropil and (**b**) number of ASYN immunolabeled neuronal cell bodies of Line 61 ASYN transgenic mice (****p < *0.0001 vs. non-transgenic/vehicle controls*). NPT200-11 administration (1 & 5 mg/kg) produced statistically significant reductions in transgenic ASYN levels in the (a) cortical neuropil (^*####*^*p* < *0.0001 vs. Line 61 ASYN transgenic/vehicle controls*), and a trend (p = 0.0523) for reduction in cortical neuropil of Line 61 transgenic ASYN levels in mice treated with 0.5 mg/kg NPT200-11. NPT200-11 administration (5 mg/kg) produced a statistically significant reduction in (b) the number of ASYN immunopositive neuronal cell bodies in the cortex of Line 61 ASYN transgenic mice (^*##*^*p* < *0.01 vs. Line 61 ASYN transgenic/vehicle controls*). Representative images obtained at 40x are shown in (c), where the scale bar represents 40 microns.

Total and PK + resistant ASYN immunohistochemistry: In accordance with previous reports showing an increased ASYN (SYN-1) immunolabeling in the CNS of Line 61 ASYN transgenic mice, there were increases in (Fig. 6a) total ASYN immunoreactivity in the cortical neuropil and (Fig. 6b) increases in the number of total ASYN immunopositive neuronal cell bodies in the cerebral cortex of vehicle-treated Line 61 ASYN transgenic mice (****p < *0.0001 vs. vehicle-treated non-transgenic group*). The total ASYN immunolabeling in the cortex of Line 61 ASYN transgenic mice was decreased by 1 and 5 mg/kg NPT200-11 (Fig. 6a) (^*####*^*p* < *0.0001 vs. vehicle-treated ASYN transgenic group*). NPT200-11 (5 mg/kg) also reduced the number of ASYN immunopositive neurons (Fig. 6b) (^*##*^*p* < *0.01 vs. vehicle-treated Line 61 ASYN transgenic group*).

**Figure 7:**
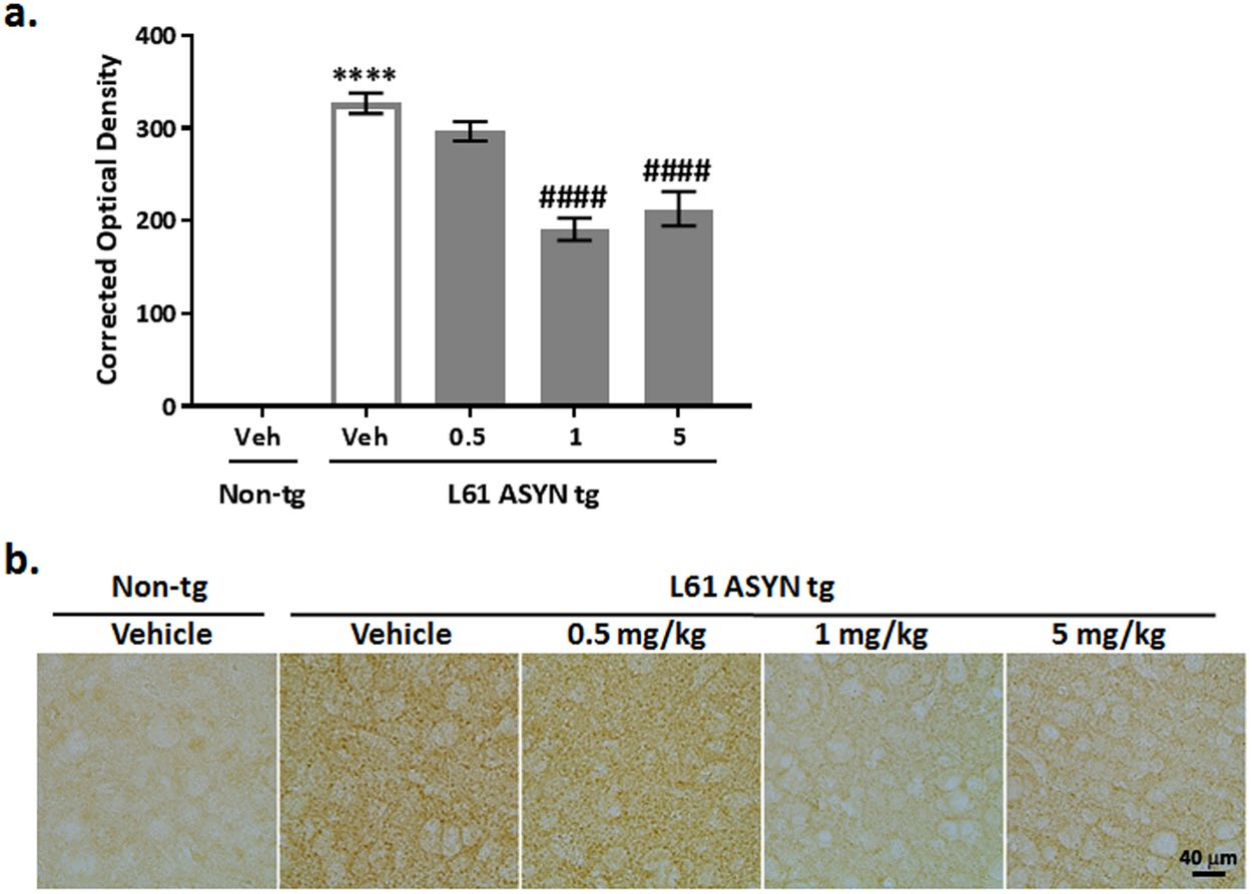
NPT200-11 administration decreased Proteinase K resistant ASYN immunolabeling in cortex of Line 61 ASYN transgenic mice. There was a statistically significant increase in PK + resistant ASYN immunolabeling in the cortical neuropil of Line 61 ASYN transgenic mice (****p < *0.0001 vs. non-transgenic/vehicle controls*). NPT200-11 administration (1 & 5 mg/kg) produced a statistically significant reduction in PK + resistant ASYN levels in the cortical neuropil of Line 61 ASYN transgenic mice (^*####*^*p* < *0.0001 vs. Line 61 ASYN transgenic/vehicle controls*). Representative images obtained at 40x are shown in (**b**), where scale bar represents 40 microns.

Proteinase K treatment (PK+) resistant ASYN immunolabeling was also increased (****p < *0.0001 vs. vehicle-treated non-transgenic group*) in the cortical neuropil of vehicle-treated Line 61 ASYN transgenic mice (Fig. 7). The increases in PK resistant ASYN immunolabeling in the cortical neuropil of Line 61 ASYN transgenic mice were decreased by 1 and 5 mg/kg NPT200-11 (^*####*^*p* < *0.0001 vs. vehicle-treated ASYN transgenic group*).

**Figure 8:**
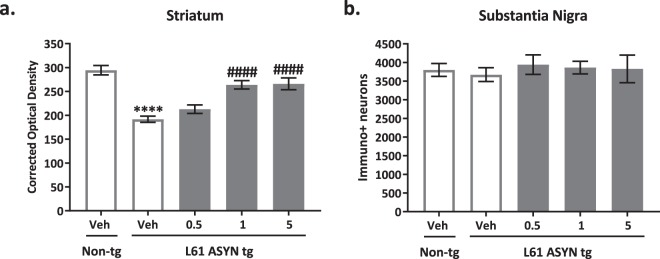
NPT200-11 administration results in normalized striatal tyrosine hydroxylase (TH) immunolabeling in Line 61 ASYN transgenic mice. There was a statistically significant decrease in tyrosine hydroxylase immunolabeling in (**a**) the striatum (****p < *0.0001 vs. vehicle-treated non-transgenic controls*), but not in the (**b**) substantia nigra of Line 61 ASYN transgenic mice. NPT200-11 administration (1 & 5 mg/kg) produced a statistically significant normalization in tyrosine hydroxylase immunolabeling levels in the (a) striatum of Line 61 transgenic mice (*1 and 5 mg/kg at* ^*####*^*p* < *0.0001 vs. vehicle-treated Line 61 ASYN transgenic controls*). There were no statistically significant effects of NPT200-11 treatment on (**b**) the number of immunopositive neuronal cell bodies in the substantia nigra.

Immunohistochemical evaluations of neurodegeneration markers: TH, NeuN and GFAP: Tyrosine hydroxylase (TH) (Fig. 8) in the striatal neuropil and cell bodies in the substantia nigra pars compact (SN) were evaluated as a measure of dopaminergic integrity and tone of the nigrostriatal pathway. There was a statistically significant reduction in TH immunolabeling levels in the striatal neuropil of vehicle-treated Line 61 ASYN transgenic mice (Fig. 8a) (****p < 0.0001 v*s. vehicle-treated non-transgenic control group*), and this reduction was ameliorated in Line 61 ASYN transgenic mice treated with 1 or 5 mg/kg NPT200-11 (^*####*^*p* < *0.0001 vs. vehicle-treated Line 61 ASYN transgenic group*). There were no phenotypic differences or treatment effects noted in tyrosine hydroxylase immunolabeling of neuronal cell bodies in the substantia nigra (Fig. 8b).

**Figure 9:**
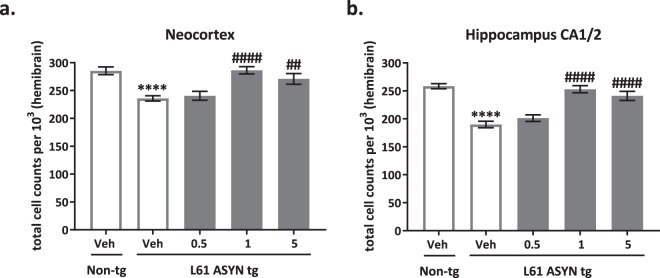
NPT200-11 administration normalizes NeuN immunopositive cell counts in cortex and hippocampus of Line 61 ASYN transgenic mice. There was a statistically significant decrease in NeuN immunopositive cell counts in the (a) neocortex (****p < *0.0001 vs. vehicle-treated non-transgenic controls*) and (b) hippocampus (****p < *0.0001 vs. vehicle-treated non-transgenic controls*). NPT200-11 administration (1 & 5 mg/kg) produced statistically significant normalizations in NeuN immunopositive cell in both regions (^*##*^*p* < *0.01 and* ^*####*^*p* < *0.0001 vs. vehicle-treated Line 61 ASYN transgenic controls*).

NeuN immunohistochemistry (Fig. 9) was evaluated as a measure of neuronal cell loss in the neocortex and hippocampal CA3 region. There were statistically significant decreases in NeuN-immunopositive cells in both the (Fig. 9a) neocortex and (Fig. 9b) hippocampus of vehicle-treated Line 61 ASYN transgenic mice (****p < *0.0001 vs. vehicle-treated non-transgenic control group, for both regions*). NeuN immunolabeling was normalized in Line 61 ASYN transgenic mice treated with 1 or 5 mg/kg NPT200-11 (^*##*^*p* < *0.01 or* ^*####*^*p* < *0.0001 vs. vehicle-treated Line 61 ASYN transgenic control group*).

**Figure 10:**
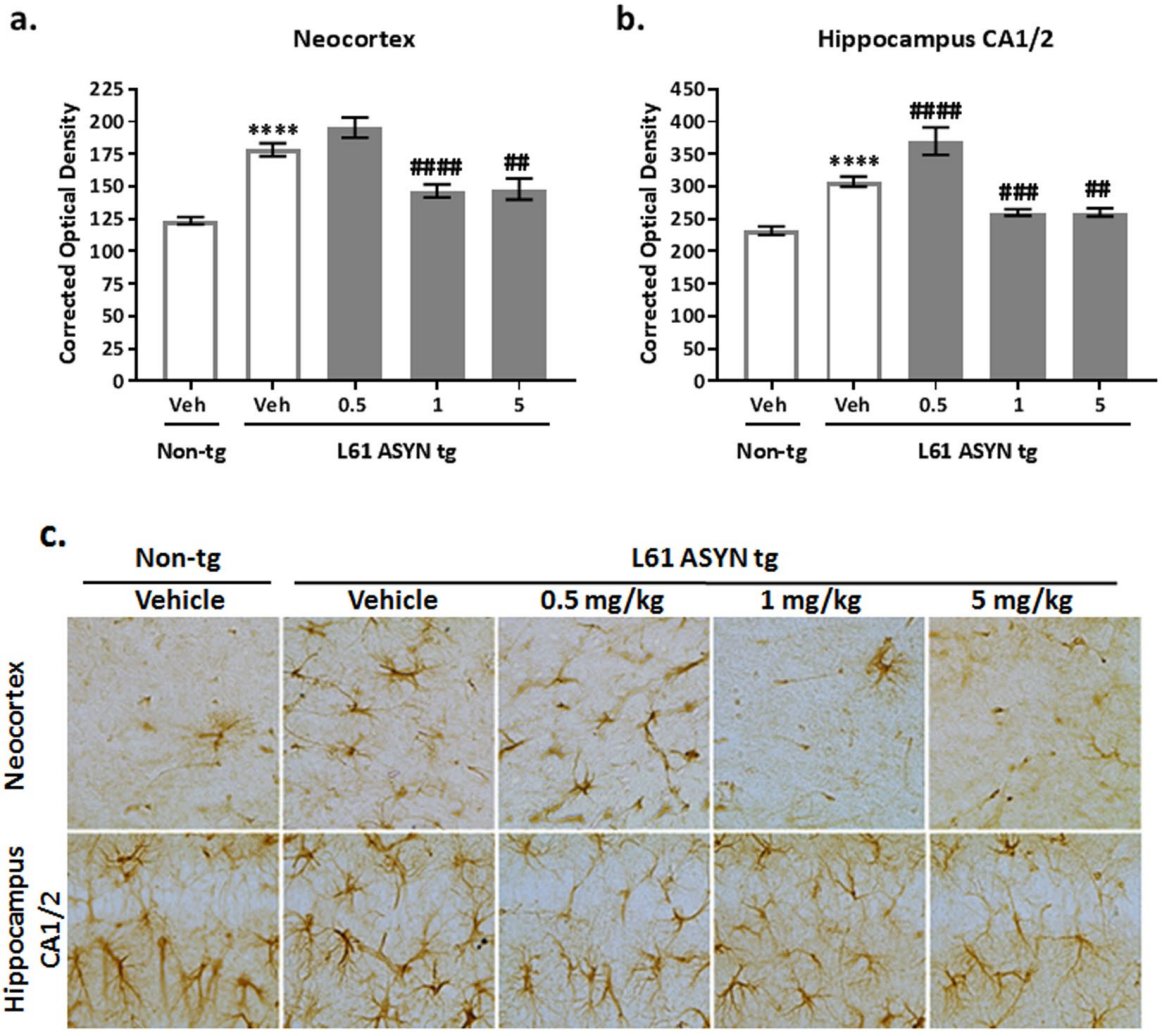
NPT200-11 treatment decreases GFAP immunolabeling in Line 61 ASYN transgenic mice. There was a statistically significant increase in GFAP immunolabeling in the (**a**) neocortex and (**b**) hippocampus CA 1/2 of Line 61 ASYN transgenic mice (****p < *0.0001 vs. vehicle-treated non-transgenic controls*). NPT200-11 administration produced statistically significant normalizations in GFAP immunolabeling in the (**a**) neocortex (1 and 5 mg/kg, ^*####*^*p* < *0.0001 and* ^*##*^*p* < *0.01 vs. vehicle-treated Line 61 ASYN transgenic controls*) and (**b**) hippocampus (1 and 5 mg/kg ^*####*^*p* < *0.0001*, ^*###*^*p* < *0.001 and* ^*##*^*p* < *0.01 and* ^*#*^*p* < *0.05 vs. vehicle-treated Line 61 ASYN transgenic controls*). An increase in GFAP levels was noted in the hippocampus of Line 61 ASYN transgenic mice treated with 0.5 mg/kg NPT200-11 (^*####*^*p* < *0.0001 vs. vehicle-treated Line 61 ASYN transgenic control group)*. Representative images obtained at 40x are shown in (**c**).

GFAP immunhistochemistry was evaluated as a measure of CNS inflammation/astrogliosis in the neocortex and hippocampal CA1/2 region. GFAP levels were increased in vehicle-treated Line 61 ASYN transgenic mice in both regions (Fig. 10) (****p < 0.0001 vs*. vehicle-treated non-transgenic control group, for both regions)*. GFAP immunolabeling in the (Fig. 10a) neocortex and (Fig. 10b) hippocampus were normalized in Line 61 ASYN transgenic mice treated with 1 or 5 mg/kg NPT200-11 (^*##*^*p* < *0.01*, ^*###*^*p* < *0.001 or* ^*####*^*p* < *0.0001 vs. vehicle-treated Line 61 ASYN transgenic control group*). An increase in GFAP levels was noted in the hippocampus of Line 61 ASYN transgenic mice treated with 0.5 mg/kg NPT200-11 (^*####*^*p* < *0.0001 vs. vehicle-treated Line 61 ASYN transgenic control group)*.

**Figure 11:**
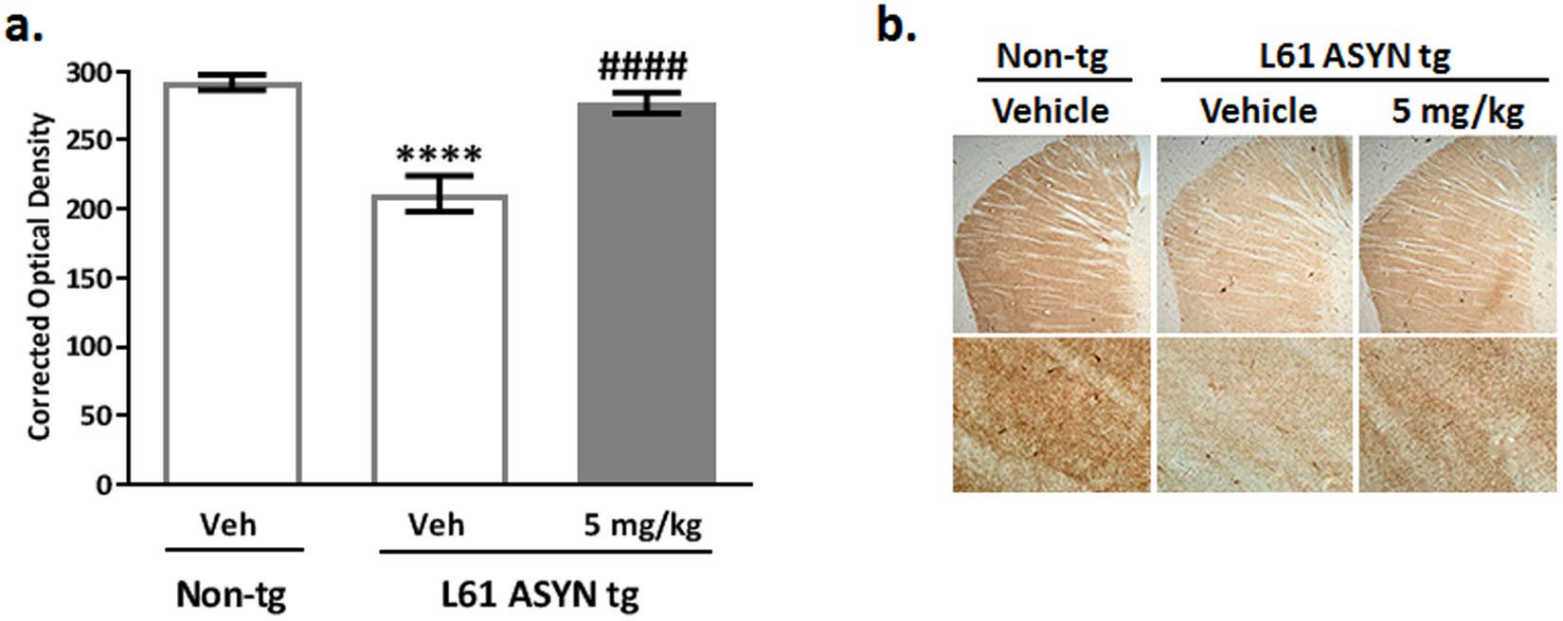
NPT200-11 treatment increases striatal dopamine transporter (DAT) levels in Line 61 ASYN transgenic mice. There was a statistically significant decrease in DAT immunolabeling (**a**) in the striatum of Line 61 ASYN transgenic mice compared to non-transgenic vehicle-treated control mice (****p < *0.0001 vs. vehicle-treated non-transgenic mice*). This phenotypic difference in the ASYN transgenic mice was reversed by NPT200-11 treatment (5 mg/kg) with normalized striatal DAT immunolabeling in Line 61 ASYN transgenic mice (^*####*^*p* < *0.0001 vs. vehicle-treated Line 61 ASYN transgenic group*). Representative low and higher resolution (40x) images are shown in panels (**b**).

Measurement of striatal dopamine transporter (DAT) immunohistochemistry: Analysis of striatal dopamine transporter (DAT) levels via immunohistochemistry (Fig. 11) was included as a potentially translatable biomarker to inform clinical development of NPT200-11. There were statistically significant decreases in DAT immunolabeling in the striatum of Line 61 ASYN transgenic mice compared to non-transgenic vehicle-treated control mice (****p < *0.0001 vs. vehicle-treated non-transgenic mice*). This phenotypic difference in the ASYN transgenic mice was robustly reduced in Line 61 ASYN transgenic mice treated with 5 mg/kg NPT200-11 (^***####***^*p* < *0.0001 vs. vehicle-treated Line 61 ASYN transgenic mice*).

## Discussion

NPT200-11 emerged from a drug-discovery effort aimed at developing an inhibitor of ASYN misfolding for the treatment of Parkinson’s disease and related disorders. As shown in these studies this clinical candidate is orally bioavailable, brain penetrating and has beneficial actions against a range of therapeutically relevant endpoints in a synucleinopathy-based animal model of Parkinson’s disease.

Numerous lines of evidence from human genetics, human pathology, cell based systems and animal models have implicated dysregulation of alpha-synuclein as an underlying driver of neurodegeneration in Parkinson’s disease and other synucleinopathies including multiple system atrophy and dementia with Lewy bodies^1–3^. Most research further suggests that misfolded and oligomeric aggregates of alpha-synuclein may be the most toxic species^19–22^. Thus a therapeutic intervention which could prevent the misfolding of alpha-synuclein and its oligomerization into toxic species may reduce neurodegeneration, neuroinflammation and the consequent disease propagation. Thus the strategy taken in this drug-discovery effort was to target specific regions of alpha-synuclein thought to be responsible for the dimerization and subsequent oligomerization into toxic conformations.

Initial lead compounds from this effort^5^ evolved from peptide sequences complementary to targeted regions on the alpha-synuclein protein thought to be important for misfolding and oligomerization into toxic (sometimes referred to a propagating) species. These initial compounds lacked metabolic stability, had low oral bioavailability and less than optimal brain penetration but nevertheless allowed validation of this therapeutic approach in animal models. The compound described in this report, NPT200-11, addressed the pharmacokinetic shortcomings of initial leads and, as shown here, maintained robust beneficial actions in synuclein-based animal models. We previously demonstrated the feasibility of repeated longitudinal retinal imaging evaluations of ASYN::GFP in the PDNG78 transgenic mouse model of DLB/PD^6^, as a method to evaluate and track the progression of neurodegenerative changes in animal models of Parkinson’s disease. Progressive pathological features in the PDNG78 retina were shown to mirror CNS pathology, thereby providing a means to non-invasively and repeatedly evaluate potential therapeutic interventions in a transgenic mouse model of PD/DLB. In the present study repeated longitudinal measures of eGFP-tagged ASYN (ASYN::GFP) in the retina demonstrated a robust, statistically significant time-dependent reduction in ASYN::GFP in the retina of NPT200-11- treated transgenic mice (5 mg/kg, IP, daily for up to 3 months). Retinal imaging in Alzheimer’s disease clinical trials has been enabled by the development of Abeta oligomer imaging ligands, however, at present there are no ASYN ligands available for retinal imaging. Nonetheless, the method (in conjunction with an appropriate mouse model such as the PDNG78 mouse) can be used to evaluate the PK/PD relationship of treatments such as NPT200-11 to ASYN::GFP.

The effects of NPT200-11 administration (0, 0.5, 1 & 5 mg/kg) on PD-relevant neuropathological and behavioral endpoints were evaluated in three month studies utilizing a transgenic mouse model of PD overexpressing human wild type ASYN under the murine Thy-1 promoter (commonly referred to as the Line 61 ASYN transgenic mice). Motor behavioral evaluations demonstrated improved performance on a measure of balance and gait (round beam traversal test). Postural instability and gait disorder are well-described clinical features of PD that respond poorly to dopaminergic treatment^23^. Neuropathological examination showed that NPT200-11 decreased the accumulation of total ASYN levels and proteinase K (PK+)-resistant ASYN aggregates in the CNS, which were accompanied by the normalization of neuronal and inflammatory markers (NeuN, tyrosine hydroxylase (TH) and glial fibrillary acidic protein (GFAP)). There were no apparent adverse effects of compound administration on subject health, and all treatments were well tolerated for the duration of the experiment. It is notable that NPT200-11 ameliorated decreases in dopamine transporter (DAT), a clinically translatable marker in the line 61 transgenic mice. Thus a 5 mg/kg dose of NPT200-11 produced consistent benefit across the most number of behavioral and neuropathological endpoints evaluated, including those which can be clinically evaluated.

Evaluation of the pharmacokinetic of NPT200-11 in blood and brains of wildtype and transgenic mice provide insights into the exposure profile of NPT200-11 that is required for efficacy in this model. These data suggest that the beneficial actions of NPT200-11 are achieved despite a relatively short half-life in plasma. This raises the possibility that repeated transient exposures, rather than sustained exposures, are sufficient to produce efficacy. Alternative explanations include the possibility of a long-lived and biologically active metabolite of NPT200-11, or that NPT200-11 is retained in the brain longer than predicted on the basis of its plasma half-life; however to date we have no evidence for accumulation of NPT200-11 in the brain (*data not shown*).

In conclusion, multiple studies of the effects of NPT200-11 in transgenic mice that overexpress wild type ASYN or ASYN linked to GFP demonstrated a wide range of beneficial actions, including: reductions in ASYN pathology, improvements in a measure of balance and gait; reduced neurodegeneration and reduced CNS inflammation. Importantly there was improvement of a clinically translatable biomarker for striatal dopaminergic integrity. These studies confirm and extend studies of a previously evaluated ASYN misfolding inhibitor, NPT100-18A^5^ with a compound possessing pharmacokinetic and safety properties suitable for clinical development. One important outcome of the current studies was the determination of effective dose levels using the various outcome measures, as this could provide guidance when selecting doses in forthcoming clinical studies.

## Electronic supplementary material

Table S1

## Data Availability

Reasonable requests for access to data generated and analyzed in this study will be considered by the corresponding author.
