# Supplementary material for: The small molecule alpha-synuclein misfolding inhibitor, NPT200-11, produces multiple benefits in an animal model of Parkinson’s disease
Source: Sci Rep. 2018 Nov 1;8:16165. doi: 10.1038/s41598-018-34490-9 (PMC6212487; doi:10.1038/s41598-018-34490-9)
Supplement: Supplementary file 1 — Table S1 [file 41598_2018_34490_MOESM1_ESM.docx]

**Supplemental Table S1:** Price, D.L, Koike, M.A., Khan A., Wrasidlo, W. Rockenstein, E., Masliah, E. & Bonhaus, D. “*The small molecule alpha-synuclein misfolding inhibitor, NPT200-11, produces multiple benefits in an animal model of Parkinson’s disease*”

**Comparison of key mouse pharmacokinetic properties of NPT100-18A and NPT200-11 for a single 10 mg/kg dose**

| **Compound ID** | **T ½**  **(hr)** | **T_max_ (hr)** | **Cmax (ng/mL)** | **Brain to plasma exposure ratios** | | | **%F** |
| --- | --- | --- | --- | --- | --- | --- | --- |
| **NPT100-18A** | **Plasma**  2.80  **Brain**  0.42 | **Plasma**  0.08  **Brain** 0.08 | **Plasma**  3843  **Brain**  40.93 | 0.03 | ND | 0.03 | 0.32 |
| **NPT200-11** | **Plasma**  1.11  **Brain**  1.75 | **Plasma**  0.25  **Brain** 0.5 | **Plasma** 2223.04  **Brain** 5279.62 | 1.99 | 0.44 | 1.05 | 53 |
| **Route of Administration** | IV | IP | IV | IV | PO | IP |  |
